# Prediction of HIV-1 protease cleavage site from octapeptide sequence information using selected classifiers and hybrid descriptors

**DOI:** 10.1186/s12859-022-05017-x

**Published:** 2022-11-08

**Authors:** Emmanuel Onah, Philip F. Uzor, Ikenna Calvin Ugwoke, Jude Uche Eze, Sunday Tochukwu Ugwuanyi, Ifeanyi Richard Chukwudi, Akachukwu Ibezim

**Affiliations:** 1 Department of Pharmaceutical and Medicinal Chemistry, University of Nigeria, Nsukka, Nigeria; 2 Department of Pharmaceutical Microbiology and Biotechnology, University of Nigeria, Nsukka, Nigeria; 3 Department of Clinical Pharmacy and Pharmacy Management, University of Nigeria, Nsukka, Nigeria; 4 Department of Pharmaceutical Technology and Industrial Pharmacy, University of Nigeria, Nsukka, Nigeria

**Keywords:** HIV-1 protease, Stratified 10-fold CV, Machine learning, Cleavage site, Octapeptide sequence, Amino acid binary profile, Physicochemical properties, Bond composition, Numerical descriptors

## Abstract

**Background:**

In most parts of the world, especially in underdeveloped countries, acquired immunodeficiency syndrome (AIDS) still remains a major cause of death, disability, and unfavorable economic outcomes. This has necessitated intensive research to develop effective therapeutic agents for the treatment of human immunodeficiency virus (HIV) infection, which is responsible for AIDS. Peptide cleavage by HIV-1 protease is an essential step in the replication of HIV-1. Thus, correct and timely prediction of the cleavage site of HIV-1 protease can significantly speed up and optimize the drug discovery process of novel HIV-1 protease inhibitors. In this work, we built and compared the performance of selected machine learning models for the prediction of HIV-1 protease cleavage site utilizing a hybrid of octapeptide sequence information comprising bond composition, amino acid binary profile (AABP), and physicochemical properties as numerical descriptors serving as input variables for some selected machine learning algorithms. Our work differs from antecedent studies exploring the same subject in the combination of octapeptide descriptors and method used. Instead of using various subsets of the dataset for training and testing the models, we combined the dataset, applied a 3-way data split, and then used a "stratified" 10-fold cross-validation technique alongside the testing set to evaluate the models.

**Results:**

Among the 8 models evaluated in the “stratified” 10-fold CV experiment, logistic regression, multi-layer perceptron classifier, linear discriminant analysis, gradient boosting classifier, Naive Bayes classifier, and decision tree classifier with *AUC*, *F-score*, and *B. Acc.* scores in the ranges of 0.91–0.96, 0.81–0.88, and 80.1–86.4%, respectively, have the closest predictive performance to the state-of-the-art model (*AUC* 0.96, *F-score* 0.80 and *B. Acc.* ~ 80.0%). Whereas, the perceptron classifier and the K-nearest neighbors had statistically lower performance (*AUC* 0.77–0.82, *F-score* 0.53–0.69, and *B. Acc.* 60.0–68.5%) at *p* < 0.05. On the other hand, logistic regression, and multi-layer perceptron classifier (*AUC* of 0.97, *F-score* > 0.89, and *B. Acc.* > 90.0%) had the best performance on further evaluation on the testing set, though linear discriminant analysis, gradient boosting classifier, and Naive Bayes classifier equally performed well (*AUC* > 0.94, *F-score* > 0.87, and *B. Acc.* > 86.0%).

**Conclusions:**

Logistic regression and multi-layer perceptron classifiers have comparable predictive performances to the state-of-the-art model when octapeptide sequence descriptors consisting of AABP, bond composition and standard physicochemical properties are used as input variables. In our future work, we hope to develop a standalone software for HIV-1 protease cleavage site prediction utilizing the linear regression algorithm and the aforementioned octapeptide sequence descriptors.

**Supplementary Information:**

The online version contains supplementary material available at 10.1186/s12859-022-05017-x.

## Background

The human immunodeficiency viruses (HIV) are a member of the lentiviruses which are distributed worldwide. They are sometimes referred to as "slow viruses" to make reference to their manner of action, since they enter the body and stay there for a long time. They have the uncanny capability of encoding information into the DNA of the host cell and of replicating in non-dividing cells [1]. HIV-1 and HIV-2 are the two forms of HIV that have been identified. The most virulent, pathogenic, and predominant strain worldwide is HIV-1. Generally, when people speak of HIV without mentioning the kind of virus, they are usually referring to HIV-1 [2]. HIV-1 protease is an essential enzyme involved in the replicative activities of HIV-1, which is responsible for the Acquired Immunodeficiency Syndrome (AIDS) [3]. AIDS is a worldwide epidemic that has resulted in approximately 25 million fatalities especially in developing nations. Poor people in developing nations are regarded to be the most vulnerable to AIDS primarily because their immune systems have been damaged by previous infections. Furthermore, the lack of access to health care and information about AIDS puts the poor in much more danger [1]. HIV-1 protease cleaves newly created Gag and Gag-Pol polyproteins to produce the mature protein components of HIV virion, which is the infectious form of the HIV-1 virus outside of the host cell [4]. HIV virions are not infectious if they do not have a competent protease enzyme. In fact, all retroviruses, including HIV-1, achieve infectious maturation of incipient virus particles by cleavage of the polyprotein precursors by the viral protease [3, 5]. Because HIV-1 protease binds to a precursor protein in octapeptide length before cleavage, vulnerable locations in a substrate are referred to as octapeptide regions, which are made up of eight amino acids in sequence [6].

Several drug molecules have been developed to inhibit substrate cleavage by HIV-1 protease. This class of drugs is popularly called "HIV-1 protease inhibitors" and they share a common motif. They have the ability to bind tightly to the HIV-1 protease making it impossible for peptide substrates to be cleaved by HIV-1 protease [7]. It is obvious that the development of specific therapeutic agents to inhibit peptide substrate cleavage by HIV-1 protease requires the proper understanding of the bond type, and amino acid composition of the cleavage site. Moreover, specificity of therapeutic agent solves most of the problems associated with drug therapy, especially untoward drug effects and toxicity [8]. Thus, the knowledge of substrate cleavage sites becomes particularly important when applying structure-based methods to design HIV-1 protease inhibitors, which begins with the identification of the potential ligand cleavage site on a target protein [9, 10]. It is worth noting that HIV-1 protease inhibitors were the first class of drugs to be developed through structure-based approach after the crystal structure of the HIV-1 protease enzyme was resolved [11, 12]. Indinavir, nelfinavir, amprenavir, saquinavir, ritonavir, lopinavir, atazanavir, tipranavir, and darunavir are typical examples of active site competitive HIV-1 protease inhibitors developed through this approach. The majority of these inhibitors, including those discovered through screening libraries, were optimized through successive structure-based method which require the knowledge of HIV-1 protease substrate specificity [13, 14]. They are highly effective against HIV and have been a key component of anti-retroviral therapies for HIV/AIDS since the 1990s [13].

Before the popularity of machine learning in computational drug development, the time-consuming and labor-intensive laboratory-based methods were the most common way of determining the substrate cleavage site targeted by HIV-1 protease [15]. However, with the growing application of computational techniques to drug design and development, a number of supervised and unsupervised machine and deep learning predictive models have been developed by several bioinformatics researchers to predict HIV-1 protease cleavage site [6]. To get an accurate prediction of HIV protease cleavage sites, two issues must be addressed. The first issue is feature vector construction, which involves creating a set of relevant features to encode octapeptide sequences, and the second is choosing an effective machine learning algorithm to assess HIV-1 protease substrate specificity [6]. Existing models approach these two issues in a variety of ways, and some typical studies are included. Rognvaldsson et al. [16] have shown that Linear Support Vector Machine (LSVM) can achieve a superior performance in predicting substrate cleavage sites by HIV-1 protease. Singh and Su [17] proposed a prediction method (ProCleSSP) in which sequence-based, structural-based, and physicochemical features are incorporated in various machine learning algorithms, including decision trees (DTs), artificial neural network (ANN), and logistic regression (LR) for the prediction of HIV-1 protease cleavage site. Singh et al. [18] proposed a novel multitask learning model for HIV-1 protease cleavage site prediction that can extract knowledge from multiple auxiliary data sources, taking into account seven different feature descriptors and four different kernel variants of support vector machines to form the optimal multi-kernel learning (MKL) model. Li and colleagues [6] also, proposed a novel positive-unlabeled learning algorithm (PU-HIV), which uses a combination of amino acid identity, co-evolutionary patterns and chemical properties for the prediction of HIV-1 protease cleavage site. Other state-of-the-art methods for the prediction of HIV-1 protease sites include: HIVcleave [19], PROSPERous [20], iProt-Sub [21], EvoCleave [22], ProCleave [23], and DeepCleave [24].

One particular advantage of these computer-aided methods over the laboratory-based method is that no prior knowledge of the substrate is needed before making predictions about the cleavage point which makes the process less cumbersome [6]. Moreover, because it reduces cost and time, the use of computers and computer software in drug development has become commonplace [25]. Furthermore, as technology and computing power increase, the accuracy of theoretical results improves dramatically, and as a result, their predictions increasingly resemble experimental outcomes [26].

Previous studies have emphasized the importance of amino acid binary features and physicochemical properties as input variables for the prediction of HIV-1 protease cleavage site [6, 14, 27]. However, to the best of our knowledge, no model has been reported or developed that uses the exact combination of octapeptide sequence information consisting of bond composition, standard physicochemical properties, and amino acid binary profile (AABP) to predict the cleavage site of HIV-1 protease. It is on this ground that we present our investigation involving the extraction and selection of these three different kinds of octapeptide features to develop and compare selected machine learning models that can predict the HIV-1 protease cleavage sites using “stratified” 10-fold cross-validation technique.

## Methods

### Datasets

In this study, four benchmark datasets consisting of 746Dataset [28], 1625Dataset [4], SchillingDataset [16], and ImpensDataset [16] were used for the machine learning predictive models building. These benchmark datasets are collections of octapeptides containing cleavage and non-cleavage sites of HIV-1 protease. The cleavage site is located between the 4th and 5th amino acid residues [16]. The 746Dataset, 1625Dataset, SchillingDataset, and ImpensDataset contain 746 (401 cleaved and 345 non-cleaved), 1625 (374 cleaved and 1251 non-cleaved), 3272 (434 cleaved and 2838 non-cleaved), and 947 (149 cleaved and 798 non-cleaved) octapeptides, respectively. These are shown in Table 1. The datasets are available at https://archive.ics.uci.edu/ml/datasets/HIV-1+protease+cleavage.

**Table 1:** Characteristics of the four benchmark HIV-1 protease datasets as contained in Rognvaldsson et al. [16]

Datasets	No. of octapeptides	No. of cleaved octapeptides	No. of non-cleaved octapeptides
746Dataset	746	401	345
1625Dataset	1625	374	1251
SchillingDataset	3272	434	2838
ImpensDataset	947	149	798

In order to reduce redundancy and improve the accuracy of our models, the four datasets were combined into one dataset and the duplicate sequences deleted using the Pandas Python library version 1.4.2 [29]. This reduced the number of data rows to 5848 (1001 cleaved and 4847 non-cleaved). Thereafter, the combined dataset was separated into two datasets based on the cleaved and non-cleaved octapeptides labels before finally removing the labels (i.e., 1 for cleaved and − 1 for non-cleaved sequences). Then, the two separate datasets were saved as different files in CSV format. This was done to ensure a clean process in the descriptor calculations.

### Creation of octapeptide sequence logos

The cleaved and non-cleaved octapeptide sequence logos were created using the Kullback–Leibler divergence, and the Heuristic clustering algorithm available in the online Seq2Logo-2.0 webserver [30]. Sequence logos are graphical representations of the information content stored in a multiple sequence alignment (MSA), and they provide a compact and highly intuitive representation of the position-specific amino acid composition of biological sequences [31, 32]. They are commonly used to depict sequence features such as binding motifs, active sites or functional units in proteins and DNAs. At each position, there is a stack of symbols representing the amino acid residues. Large symbols represent frequently observed amino acids, large stacks represent conserved positions, and small stacks represent variable positions [39]. Unlike the conventional Shannon sequence logo that favors only the enriched amino acid residues or bases, the Kullback–Leibler sequence logo clearly highlight both enrichment and depletion of elements at each position in a sequence [33]. Enriched elements are shown on the positive y-axis while the depleted elements are shown on the negative y-axis.

### Feature extraction/vector construction

The process of converting raw data into mathematical objects that can be understood by machine learning algorithms while retaining the information in the original dataset is referred to as feature extraction or vector construction [34]. As previously said, large dimensions can cause two issues: information redundancy or noise, as well as dimension disaster. As a result, feature selection is critical in classification tasks [35]. The number and type of peptide feature descriptors selected determine to a great extent the performance of a model [6]. Amino acids are the building blocks of peptides and proteins. There are 20 distinct amino acids, each with its own set of properties. The diversity and uniqueness of a protein's structure and function can be influenced by the properties of individual residues within the protein [18]. Here, we use a combination of 14 standard physicochemical properties, 160 amino acid binary profiles (AABP) and 4 bond compositions obtained using the peptide features extraction algorithms implemented in Pfeature Python package [36] to construct a 178-dimension (178D) feature vector. Thereafter the datasets for both the cleaved and non-cleaved octapeptides features were combined into one data frame after codifying the cleaved octapeptide as “1” and non-cleaved octapeptides as “0” using the mapping functionality implemented in Python 3 [37]. In order to eliminate low variance features and avoid dimension disaster, the variance feature selection algorithm of Scikit-learn version 1.0.2 [38] was applied on the 14 standard physicochemical properties and 4 bond compositions feature vectors at a threshold value of 0.0, which keeps all features with non-zero variance. That is, characteristics with the same value in all samples are removed [38]. However, the algorithm returned 18 features indicating that there are no features with the same value in the standard physicochemical properties and bond compositions. Considering that the 160 AABP features are a one-hot encoding of the octapeptides, it was unnecessary to perform feature selection on them [27]. The CSV file of the 178D constructed octapeptide descriptors is available in the Additional file 2: 178D octapeptide sequence descriptors calculated from Pfeature software. The features are further explained below.

- Bond composition
  Atoms and the bonds that connect them make up all amino acids. Aromatic, hydrogen, single, and double bonds are the four types of bonds considered in this work. The bond composition was calculated using the following formula:
  $$BT{C}_{i} = \frac{{B}_{i}}{N}$$
  where $BT{C}_{i}$ is bond composition for bond of type i, ${B}_{i}$ and $N$ are respectively, the number of atoms of type i and number of atoms in an octapeptide.
- AABP
  The usefulness of binary profiling while building machine learning predictive models has been demonstrated in several studies [27]. AABP contains information on the composition as well as order of amino acids present in a protein or peptide [39]. For each octapeptide, amino acid binary profiles were generated, with each amino acid represented by a 20-dimensional vector. This is the so-called orthogonal encoding scheme, where each amino acid is represented by an orthogonal vector of 20 bits. For instance, Alanine (A) corresponds to 10,000,000,000,000,000,000, and Tyrosine (Y) corresponds to 00,000,000,000,000,000,001. For each octapeptide sequence with length $n$, where $n=8$, the total dimension of the binary feature vector is $20\times n$, resulting in each octapeptide being mapped to a 160-dimensional vector in a sparse orthogonal representation [40].
- Standard physicochemical properties
  14 standard physicochemical properties were used to represent each octapeptide sequence. Using the standard conversion formula, the values of each Physicochemical property for all 20 amino acids were normalized between 0 and 1 [36]. Each scalar value in the input vector represents the average value of a distinct physicochemical property of residues. Table 2 gives the list and description of the standard physicochemical property features extracted. We used the following formula to calculate these features:
  $$PC{P}_{i} = \frac{{P}_{i}}{L}$$
  where $PC{P}_{i}$ is Physico-chemical properties composition of type i, ${P}_{i}$ and $L$ are sum of property of type i, and length of sequence, respectively.

**Table 2:** Description of the standard physicochemical properties employed for the models training

Physicochemical property	Abbreviated representation in pfeature	Description
Positively charged	PCP_PC	A measure of the residual positive charge resident on an octapeptide
Negatively charged	PCP_NC	A measure of the residual negative charge on an octapeptide
Neutral towards water	PCP_NE	Answers the question, how neutral is the octapeptide towards water
Polarity	PCP_PO	A measure of how polar an octapeptide is
Non-polarity	PCP_NP	A measure of how non-polar an octapeptide is
Aliphaticity	PCP_AL	Gives the measure of how aliphatic the octapeptide is
Cyclicity	PCP_CY	Gives a measure of how many cyclic rings are present in an octapeptide
Aromaticity	PCP_AR	Answers the question, how aromatic is the octapeptide?
Acidity	PCP_AC	Gives a measure of how acidic an octapeptide is
Basicity	PCP_BS	Gives the measure of how basic an octapeptide is
Neutral pH	PCP_NE_pH	Measures the pH of an octapeptide under neutral condition
Hydrophilicity	PCP_HB	Measures the tendency of water to include non-polar molecules
Hydrophobicity	PCP_HL	Measures the tendency of water to exclude non-polar molecules
Sulfur Content	PCP_SC	Gives the proportion of Sulfur in an octapeptide

### Machine learning algorithms

**Fig. 1:**
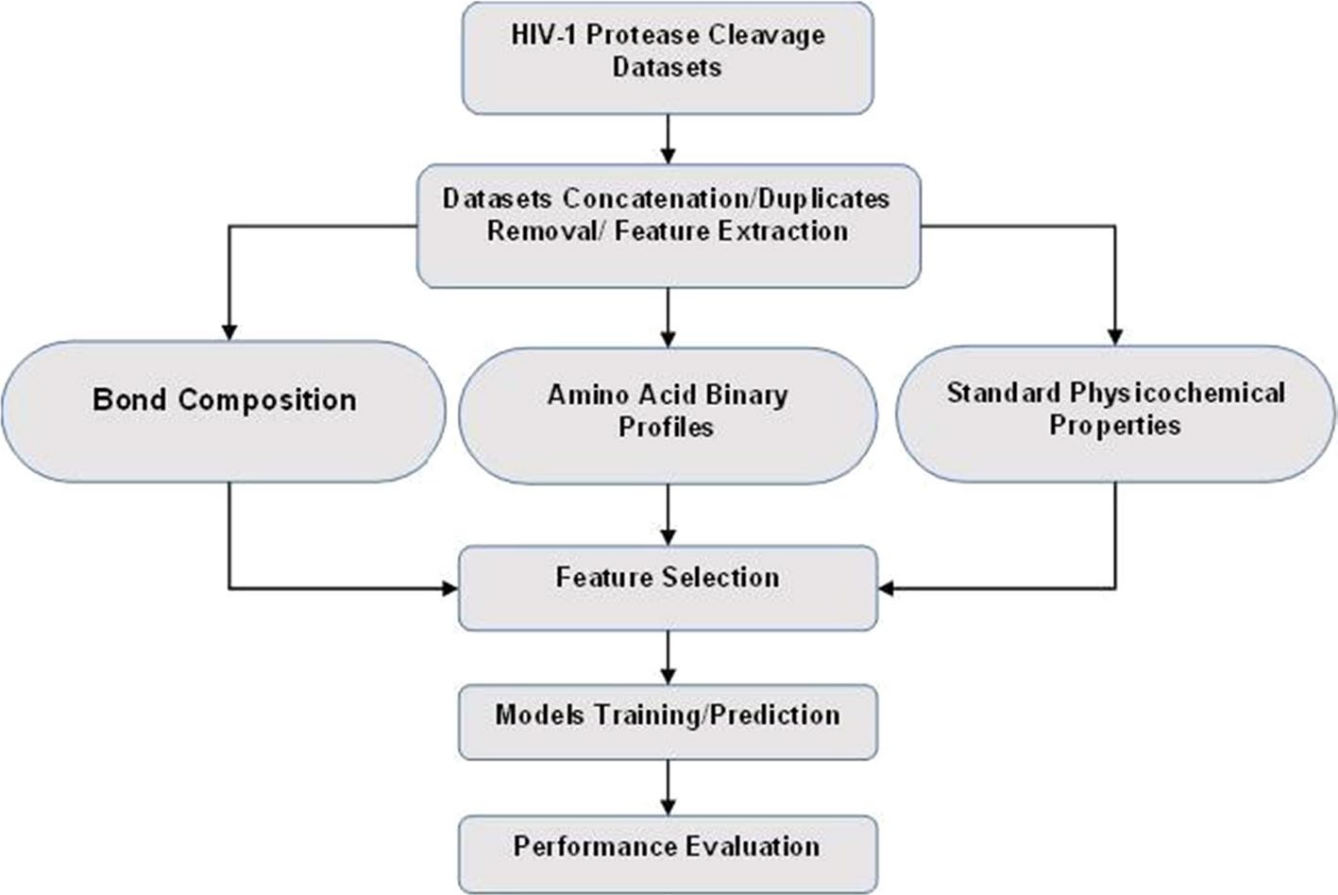
The workflow of the models building methodologies employed in the study

Eight commonly utilized supervised machine learning algorithms namely: linear discriminant analysis (LDA), gradient boosting classifier (GBC), k-nearest neighbors (KNN), Naive Bayes classifier (NBC), decision tree classifier (DTC), multi-layer perceptron classifier (MLPC), perception classifier (PC) and logistic regression (LR) were selected for this study. These models are known to perform well in binary classification problems by finding an optimal classification hyperplane that maximizes the interval between different types of samples in a given dataset [41]. The models were trained using the 178D feature vectors. Linear support vector machine (LSVM) was chosen as the standard to compare the performance of our models owing to the literature report as the state-of-the-art classification technique for HIV-1 protease cleavage site prediction [16]. Generally, Support Vector Machine (SVM), invented by Vapnik in 1995, is a well-known supervised machine learning algorithm for binary classification problems that are based on statistical learning theory. SVM reduces classification errors by determining the separation hyperplane using support vectors which transform sample data into a higher-dimensional space using the kernel function. Four fundamental kernel functions: linear, polynomial, sigmoid, and radial basis functions are typically employed [42]. The workflow of the Models building methodologies is shown in Fig. 1.

### Model training, performance evaluation and data visualization

Prior to feature selection, the dataset was split into three distinct sets as follows: validation set (10%), training set (70%), and testing set (20%). This three-way data split procedure ensures that we keep overfitting and overestimation in check. The validation set was used for tuning of the hyper-parameters of the models via the exhaustive 10-fold GridSearchCV algorithm and manually where applicable. Once the optimal values of the hyper-parameters were obtained, the models were fitted with the training dataset. Also, the “stratified” 10-fold CV experiment was performed using the training set. Thereafter, the predictive performances of the trained models were further evaluated on the testing set, which served as an external dataset used only for the purpose of re-evaluating the real performance of our models. The three most extensively used CV methods in machine and statistical learning are $K$-fold, Monte-Carlo, and leave-one-out cross-validation [35, 43]. $K$-fold cross-validation is a method of model fine-tuning by resampling the dataset into training and validation k times. That is, the dataset is partitioned into $k$ mutually exclusive subsets or folds, say ${D}_{1}, {D}_{2}, {D}_{3}, \dots , {D}_{k-1}, {D}_{k}$ of roughly equal size each. Training and testing are repeated $k$ times. In iteration $i$, partition ${D}_{i}$ is reserved as the test set, and the remaining partitions are collectively used to train the models. This process is repeated until each fold is used as a testing set [44, 45]. For classification, the performance metric is evaluated on each fold, and the average is used as a measure of the model's overall performance [44]. This reduces the bias and variance errors in our models, which could lead to underfitting and overfitting, respectively [46]. For an imbalanced dataset, the $K$-fold cross-validation is "stratified", which ensures proportionate distribution of the negative and positive examples among the folds[44]. On the other hand, data visualization was performed with matplotlib version 2.2 [47] and seaborn version 0.11.2 [48] Python Libraries.

Five common performance metrics—*balanced accuracy*, *AUC*, *sensitivity*, *specificity*, and *F-score*—that have been identified by the literature [49–51] as being significant were used to thoroughly assess the performance of our models. The confusion matrix was also computed using the testing set. The confusion matrix is available in the Additional file 1: Fig. S1—Confusion matrix of our models using the testing set. Given the imbalance in our dataset, we used balanced accuracy (*B. Acc.*) to avoid overestimation of performance on imbalanced datasets (as is the case here). It is the raw accuracy or the macro-average of recall scores per class, where each sample is weighted according to its true class's inverse predominance. As a result, for balanced datasets, the score equals accuracy. Balanced accuracy is equal to the arithmetic mean of recall (sensitivity) and specificity when considering a binary class. Sensitivity (*Sen.*) and specificity (*Spec.*), which respectively measures how well a model classifies positive examples (cleaved octapeptide sequences) as positive and negative examples (un-cleaved octapeptide sequences) as negative, were included. The decision to classify cleaved octapeptide sequences as "positive examples" and un-cleaved octapeptide sequences as "negative examples" is essentially methodical, giving the terms "sensitivity" and "specificity" as employed here meaning. The F-index (*FI*) was used to provide a single metric that addressed both precision and recall. It can be thought of statistically as a weighted harmonic mean of precision and specificity. The area under the receiver operating characteristic (ROC) curve, abbreviated as AUC, was also used. Because it is non-parametric and threshold independent, the AUC is one of the most appropriate measures of performance. It is derived from the ROC curve, which is a key metric for model evaluation. The ROC curve is a graph that plots the true positive rate (i.e., sensitivity) against the false positive rate (i.e., 1-specificity) for various parameter cutoff points. They all range in value from 0 through 1, with 1 as the best achievable value and 0 as the worst. These metrics are defined by the following relationships, where *TP*, *TN*, *FP*, and *FN* denote the number of true positives, true negatives, false positives, and false negatives, respectively.

$$Sen. = \frac{TP}{TP + FN}$$

$$Spe. = \frac{TN}{TN + FP}$$

$$B. Acc. =\frac{1}{2}( Sen. + Spe.)$$

$$F{\text{ - }}Index = 2\frac{{Precision\; \times \;Spe.}}{{Precision\; + \;Spe.}}$$

An additional metric called Jaccard index (*JI*), introduced by Grove Karl Gilbert in 1884 for comparing the similarity and variability between two sets [52] was used. It is commonly used in problems involving binary classification to compute the similarity between the predicted values and the actual values [53]. The Jaccard index also ranges from 0 to 1, with 0 indicating no overlap and 1 representing complete overlap. It is calculated with the following formula:

$$JI=\frac{{|y}_{true} \bigcap {y}_{pred}|}{|{y}_{true} \bigcup {y}_{pred}|}$$

where ${y}_{true}$ is the set of actual values and ${y}_{pred}$ is the set of predicted values.

## Results and discussion

In this study, we have been able to successfully develop and evaluate the performance of 8 machine learning models for the prediction of HIV-1 protease cleavage site via a combination of various machine learning and deep learning algorithms and selected octapeptide feature descriptors. We went further to build the SVM using the “*linear kernel*” as a standard for our models. In order to effectively evaluate the performance of our models, we adopted a diverse range of standard performance metrics using the “stratified” 10-fold CV technique and then the results compared to those of the SVM as a state-of-the-art classifier algorithm for HIV-1 protease cleavage site prediction [16]. We have explained that the essence of the stratification was to ensure that the positive and negative examples are distributed uniformly among the 10 folds during the 10-fold CV trials [44]. The values of the performance metrics we reported from the 10-fold CV are the mean of the values computed in the loop. This approach can be computationally excursive, but does not waste too much data [45]. Furthermore, using diverse cross-validated performance metrics is considered good practice and can objectively reveal the true performance of a model rather than depending on just one metric that could be biased towards a subset of the dataset. This approach reduces the risk of overfitting [6, 18, 35]. Our models performance was also evaluated on an independent testing set which was not previously exposed to the models to give a true account of the models predictive strength. Furthermore, the sequence logos of our datasets were generated to better visualize and understand the frequency and preference of amino acid residues residing at the cleavage site of the octapeptide sequences in both the cleaved and un-cleaved examples.

### Hyper-parameter tuning and training of the models

Preceding the models' training and performance evaluation, their hyper-parameters were carefully adjusted by executing an exhaustive search over each estimator's designated parameter values on the validation set. The *maximum iteration*, *tolerance*, and the *inverse of regularization strength* (*C*) of the LSVM were discovered to be optimal at 10,000, 1e−1, and 5 respectively. Typically, various logarithmic scale values of *C* (1e−5, 1e−4, 1e−3, …, 1, 10, 100, …) were tried before fine tuning it at finer granularity within a specific interval. Turning *C* correctly is considered a vital step in best practice in the use of SVMs [54], as structural risk minimization is partly implemented by an optimal value of *C*. In fact, a lower value of *C* ensures correct classification of the training data points [55, 56]. The *tolerance* of the LDA model was also discovered to have an optimal value of 1e−5 when using the singular value decomposition (*SVD*) algorithm, which is suggested for a dataset with a lot of features. The *maximum depth*, *complexity*, *learning rate*, and *tolerance* parameters of the GBC were found to be optimal at 1.0, 0.0, 1.0, and 1e−10, respectively. Meanwhile, the *number of boosting stages* to perform was optimal at 800. Since gradient boosting is fairly robust to overfitting, a large number of boosting stages usually results in better performance [57]. For the KNN classifier, the optimal value for the number of neighbors (*k*), the number of parallel fit and predict jobs (*n jobs*), the *leaf size*, and the power parameter (*p*) were found to be 4, − 1, 40, and 2, respectively. Each point in every neighborhood of the employed “*Minkowski*’ space was rated using the "*distance*" weight function. The − 1 value of the “*n jobs*” ensured that all processors were used for neighbors search. Employing the “*adam*” optimization algorithm, “*adaptive*” learning rate, ‘*rectified linear unit*’ activation function, and tuning the *hidden layer sizes*, *tolerance*, *alpha*, *maximum iteration*, and *epsilon* parameters of the MLPC to 100, 1e−4, 0.0001, 200, and 1e−8 gave optimal performance. The stochastic gradient-based optimizer (*Adam*) works well for relatively large datasets like ours in terms of both training time and validation score. The NBC model performs best when the *alpha* parameter was zeroed, which guarantees that there was no smoothing. The *C*, *tolerance*, and *maximum iteration* of the LR model were found to have optimal values of 0.99, 1e−4, and 150, respectively when using the "*l2*" penalty or regularization term, the "*lbfgs*" solver, and “*n jobs*” of − 1. The *alpha*, *maximum iteration*, and *tolerance* parameters of the PC were similarly tuned to 1e−5, 2000, and 1e−8 when using the "*l2*" regularization term and “*n jobs*” of − 1. Analogous to the GBC, adjusting the DTC's “*complexity*” parameter to 0.0, and applying the "*gini*" criterion and the "*best*" splitter achieved optimal performance.

The confusion matrix of the models was computed from the testing set using the Sci-kit learn confusion matrix display tool. This confusion matrix gives an overview of the performance of our models and further reveals the high imbalance between the positive and negative examples in our dataset, with the former being about one fourth the latter. And to account for this imbalance, we applied the balanced accuracy metric (among other metrics) instead of the normal accuracy score, which does not put class imbalance into consideration. The F1 score metric was also applied for similar reasons. Another metric called Jaccard-index, popular in the literature for measuring group overlap index [43], was also included in this study. Table 3 shows the performance of our models on the testing set, alongside the 10-fold CV across the various evaluation metrics employed in the study.

**Table 3:** Predictive performance metrics of our models

Model	Performance on testing set						10-Fold CV					
	*B. Acc*	*AUC*	*Spec*	*Sen*	*F-Index*	*JI*	*B. Acc*	*AUC*	*Spec*	*Sen*	*F-Index*	*JI*
LSVM	92.8	0.97	0.93	0.92	0.88	0.80	79.2	0.96	0.94	0.64	0.80	0.70
LDA	88.4	0.96	0.96	0.80	0.88	0.80	84.7	0.95	0.95	0.74	0.85	0.75
GBC	89.8	0.97	0.97	0.83	0.90	0.82	80.1	0.91	0.95	0.65	0.81	0.71
KNN	69.0	0.78	0.91	0.47	0.69	0.57	68.5*	0.77	0.91	0.46	0.69	0.57
NBC	87.0	0.94	0.97	0.77	0.88	0.79	83.0	0.90	0.92	0.74	0.84	0.77
DTC	73.6	0.75	0.90	0.57	0.73	0.59	75.9	0.76	0.92	0.60	0.75	0.64
MLPC	92.0	0.97	0.95	0.89	0.90	0.82	87.5	0.96	0.95	0.75	0.86	0.78
PC	50.3	0.89	1.00	0.00	0.46	0.42	60.0*	0.82	0.85	0.35	0.53	0.46
LR	90.9	0.97	0.97	0.84	0.91	0.83	86.4	0.96	0.97	0.76	0.88	0.79

Values represented in the 10-Fold Experiment table are the averages across the 10-folds; *Significant at *p* < 0.05

### 10-Fold CV performance evaluation

Comparative analysis of our models' performances following the "stratified" 10-fold CV experiment revealed that MLPC, LR, LDA, NBC, and GBC have the closest performance to the LSVM (see Table 3). Metrics which offset imbalance in datasets also agrees with this conclusion as they all have *B. Acc., F-score, JI* and *AUC* greater than 84.0%, 0.80, 0.70, and 0.90, respectively when compared to those of the LSVM with *B. Acc. score, F-score, JI* and *AUC* respectively equal to 79.2%, 0.80, 0.70, and 0.96. This supports earlier studies that LR and MLPC show high predictive capacity when diverse types of features are integrated as input features for cleavage site prediction [13, 24]. On the other hand, KNN, DTC, and PC had lower performance across the 6 standard metrics employed when compared to those of LSVM. Their *AUC, F-score, B. Acc., and JI* fall in the ranges of 0.76–0.82, 0.53–0.75, 60–75.9%, and 0.46–0.64, respectively. Singh and Su [13] have previously reported that LR performs significantly better than DTC when different types of octapeptide sequence-based, structure-based, and physicochemical descriptors are combined as input features for the task of predicting the cleavage status of an octapeptide. Based on this result, we can group our models into two distinct categories consisting of *well-performed*, and *moderately-performed* models with the MLPC, LR, LDA, NBC, and GBC belonging to the *well-performed* class, while KNN, DTC, and PC belong to the *moderately-performed* class.

The *Sen*. and *Spec*. scores, which respectively measure how well our models can categorize a cleaved octapeptide sequence as "cleaved" and an un-cleaved octapeptide sequence as "un-cleaved", followed a typical pattern as was anticipated. To quantify the gap between the two scores, the difference between the *Sen.* and *Spec.* scores of each model was estimated by subtracting the *Sen. score* from the *Spec. score* and expressing it as a percentage (see Table 4). On average, among the 9 models, the *Spec*. scores was 32.93% higher than the *Sen.* values. However, some models, such as PC and KNN, displaying differences more than 54.0%, make this discrepancy appear to be more apparent. This noticeable disparity between the *Spec.* and *Sen.* scores can best be explained by the differential distribution of positive and negative cases in our dataset. Additionally, because there are so many more negative instances, the learning algorithms tend to catch more of their behavior, which causes the *Spec.* scores to be higher than the *Sen.* scores. However, MLPC, LR, NBC, and LDA were more robust to the effect of this class imbalance, reflecting in their higher *Sen.* scores compared to the other models. In fact, the average difference in the values of their *Sen.* and *Spec.* scores is roughly 20.0%. Singh and colleague [13] also reported the effect of this class imbalance on the sensitivity of their ProCleSSP model.

**Table 4:** Percentage difference in sensitivity and specificity with the calculated *p* values of each model’s balanced accuracy against the LSVM in the 10-fold CV

Models	Difference between *Sen.* and *Spec.* in the 10-fold CV (%)	Difference between *Sen.* and *Spec.* on testing set (%)	*p* value
LSVM	31.91	1.09	1.0000
LDA	22.11	20.00	0.2084
GBC	31.58	16.87	0.6764
KNN	49.45	93.62	0.0122
NBC	19.57	25.97	0.4922
DTC	34.78	57.89	0.2388
MLPC	21.05	6.74	0.1193
PC	58.82	100.00	0.0037
LR	21.65	13.40	0.0799

**Fig. 2:**
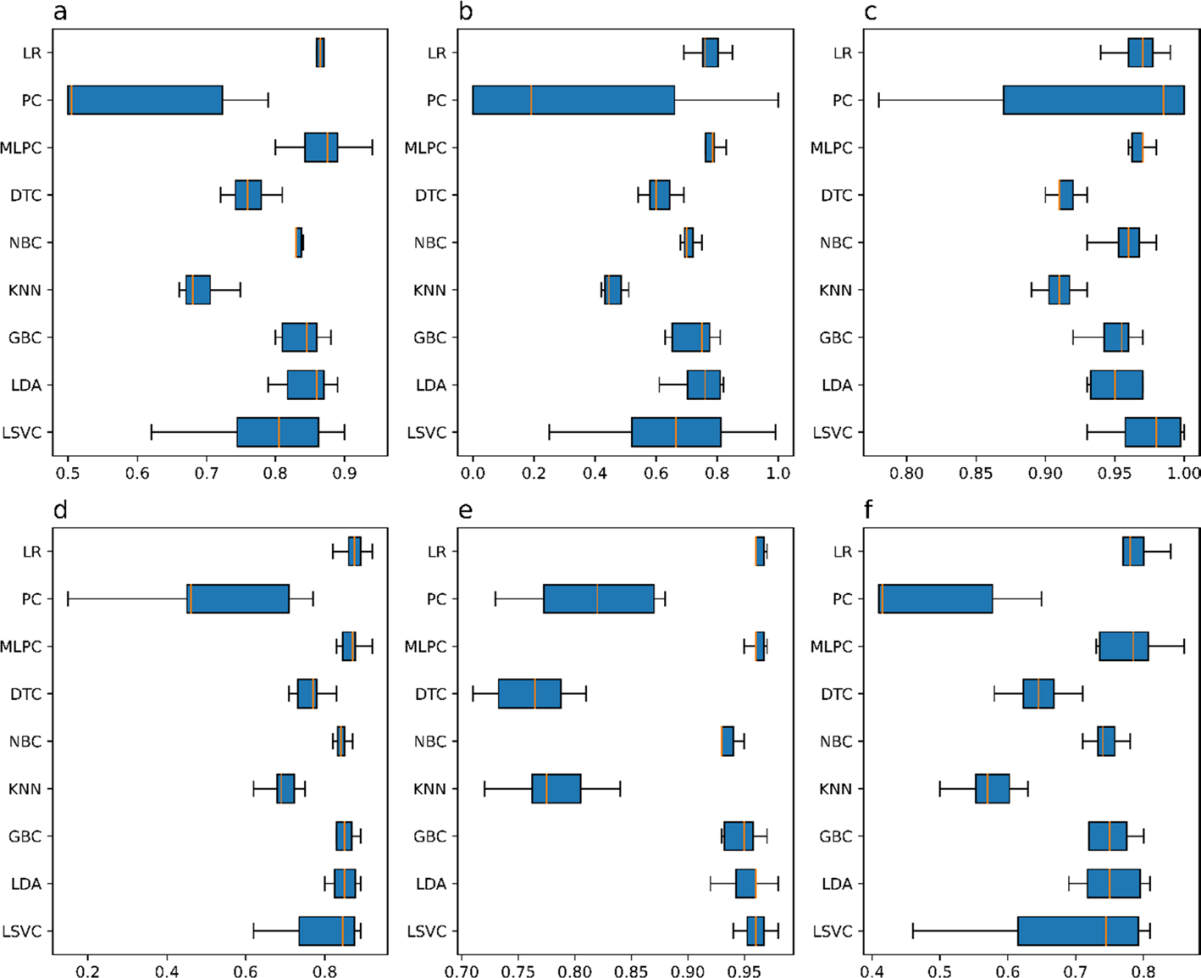
Distribution of the performance metrics scores of the models in the 10-fold CV experiment for each of the 6 standard tests conducted. **a** Balanced Accuracy Scores; **b** Sensitivity Scores; **c** Specificity Scores; **d** F-score; **e** AUC; and **f** Jaccard Index Scores

Figure 2 depicts the distribution of each model's scores across the six performance metrics as boxplots, with the lengths of the bars indicating how widely distributed the scores are for a given metric and thus directly correlate with the variation of each individual score across the ten folds. This chart thoroughly examines the 10-fold CV experiment rather than focusing solely on average scores, which may obscure some interesting conclusions. The chart clearly shows that the LR, MLPC, NBC, GBC, and LDA all have roughly similar distribution to the LSVM, whereas the PC, DTC, and KNN have more variation across the metrics, with the PC having the most variation.

To confirm this observation, we carried out a series of pairwise comparison by performing the Mann–Whitney U test on the *B. Acc.* scores of each model versus the state-of-the-art model to see if the difference in the model's accuracy is statistically significant (*p* < 0.05). *B. Acc.* scores were chosen because they are the average of sensitivity and specificity and thus provide a more reliable representation of the model's performance. The results of this test support the conclusion that there is a statistically significant difference in performance between the PC and the standard, with a *p* value of 0.0037 (see Table 4). With a *p* value of 0.0122, the KNN model also demonstrated a significant difference in performance. However, there was no statistically significant difference in the accuracy scores of the LR, MLPC, NBC, GBC, LDA, and LSVM, confirming our previous claim.

### Performance of the models on the testing set

There is a general consensus between the performance of the models in the “stratified” 10-fold CV experiment and their performance on the testing set (see Table 3). That is, in the 10-fold CV, models that performed well in terms of prediction also scored satisfactorily on the testing set, and vice versa. The behavior of the models toward different subsets of the dataset corresponds, showing, among other things, consistency and reliability in their predicted performances. However, compared to what was seen in the 10-fold CV experiment, the *B. Acc.* of the LSVM on the testing set (92.8%) was slightly higher than that of both MLPC (92.0%) and LR (90.9%). Among other things, this shows the superiority of the LSVM to classify unseen dataset better.

The percentage difference between sensitivity and specificity in the 10-fold CV experiment was substantially higher than that of the testing set, except for the PC and the KNN with differences of 93.62% and 100%, respectively. This observation may be partly explained by the fact that the additional division of the training set into 90% for training and 10% for testing in each fold of the 10-fold CV further decreased the number of positive cases in the test. In general, it is clear that the models' performance on the testing set is marginally superior to that of the 10-fold CV trial due to similar reasons. For biased classification tasks, the PC and KNN models with very high specificity (greater than 90%) and low sensitivity (less than 50%) could be very useful. That is, if we want to build a classifier that can only predict un-cleaved octapeptide sequences, the PC and KNN are good choices because they both have a bias toward un-cleaved octapeptide sequences. Finally, it is worth noting that the PC was the most reactive model towards the un-cleaved octapeptide sequences.

**Fig. 3:**
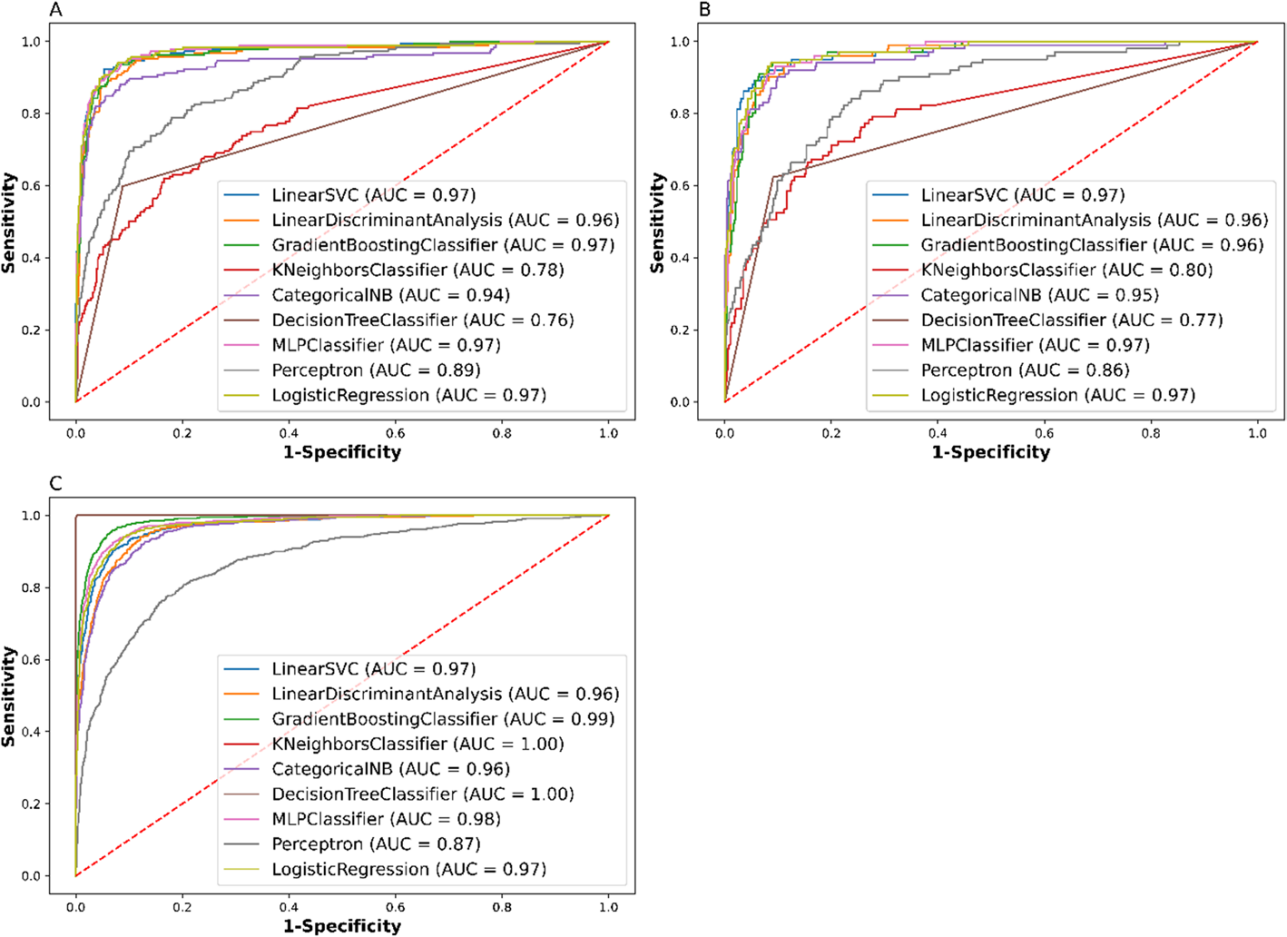
ROC curves of the models generated from **A** the testing set; and **B** the validation set; and **C** training set

Figure 3 shows the ROC curves of the models generated from various subsets of the dataset. As expected, all the model tested on the training set had very high AUCs compared to those of the validation and testing set. This makes sense given that the models were already accustomed to the training dataset during the models’ fitting.

### Comparison with existing methods

Next, we did a brief comparison between the performance of our *well-performed* models and some previously reported methods. The study by Singh and Su [13] showed that the LR and the ANN (A neural network model similar to MLPC) have accuracy and AUC > 80.0% across the four benchmark datasets using the constructed physicochemical, sequence-based and structure-based descriptors. Also, their ProCleSSP model achieved better performance accuracy of 95.1% and AUC of 0.992 in the 1625 benchmark dataset. This supports our findings about the predictive accuracy of the MLPC and LR models. Singh et al. [18] reported that their MKL model which combines seven different feature descriptors and four different kernel variants of support vector machines achieved an average accuracy of 89.12% and an average AUC of 0.982 across the 4 benchmark datasets. Nanni et al. [58] proposed a method that is based on ensembles of classifiers which gave an average 10-fold cross-validated AUC of 0.98. Li et al. [59] created a theoretical framework based on the kernel-based manifold learning method (KML) for dimensionality reduction and SVM for HIV-1 protease cleavage prediction. In a fivefold CV experiment, this KML-SVM method achieved an accuracy of 97%. Although, our method achieved lower performance accuracy compared to the aforementioned approaches, but showed consistency and reliability as it yielded similar results both in the 10-fold CV experiment and on an independent dataset (Table 3).

### Feature selection by gradient boosting algorithm

**Fig. 4:**
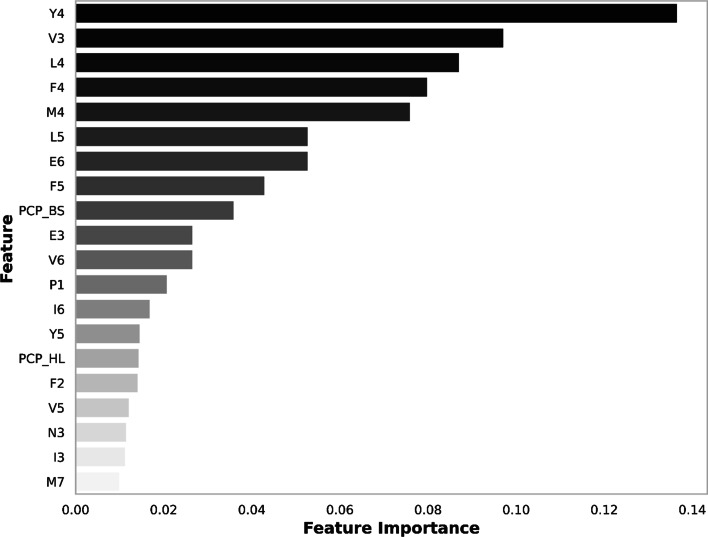
Gini importance chart of the best 20 features selected by the GBC

Figure 4 ranks the variable importance of the GBC by reduction in Gini index for the training set. Among the three octapeptide feature vectors constructed, amino acid binary profiles seem to contribute most to the predictive ability of our models, with Y4 (position 4 tyrosine) contributing most, followed by V3 (position 3 valine), L4 (position 4 leucine), F4 (position 4 phenylalanine), M4 (position 4 methionine), L5 (position 5 leucine), E6 (position 6 glutamic acid), and F5 (position 5 phenylalanine). An interesting trend is observed here, with the top-ranked amino acid residues clustering around positions 4 and 5 in the octapeptide sequences. This observation is not unexpected as the centered positions 4 and 5 in the octapeptide had previously been shown to be essential in identifying cleavage sites, and these two positions have also been shown to be relevant in the conservative classification of HIV-1 protease substrates [4]. Several physicochemical properties of the octapeptide also appear to play significant roles in the prediction of the cleavage site of HIV-1 protease. In order of importance, these physicochemical features include PCP_BS (basicity), and PCP_HL (hydrophilicity). The top-ranked hydrophilicity agrees well with the findings that water accessibility served as a discriminative factor to predict cleavage sites [54]. This supports the claim that our method can discover essential biological and chemical characteristics that can be used to find cleavage sites.

### Amino acid compositional analysis

**Fig. 5:**
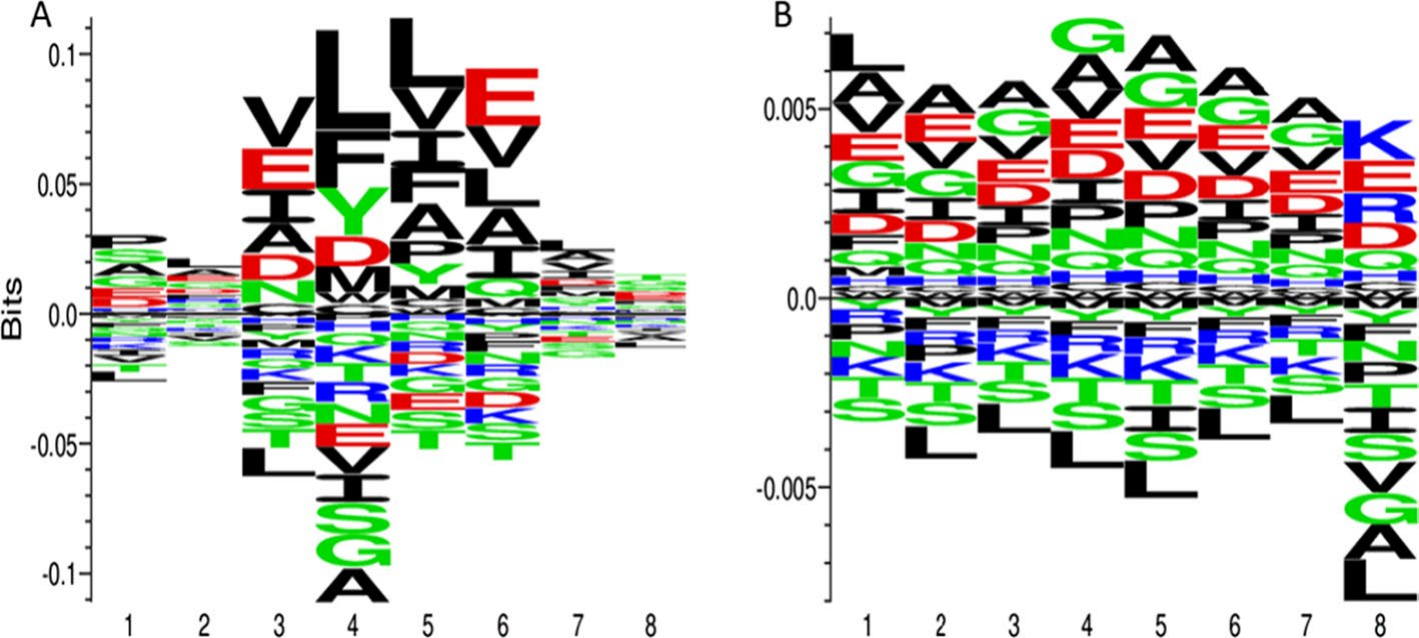
The sequence logos of **A** cleaved octapeptide; **B** un-cleaved octapeptide sequences generated with the online Seq2Logo webserver using Heuristic clustering algorithm, pseudo count with a weight of 200 and logotype as Kullback–Leibler. Enriched amino acids are shown on the positive y-axis and depleted amino acids on the negative y-axis

Before the feature selection analysis, we investigated which type of amino acid residues are preferred at specific positions in both the cleaved and un-cleaved octapeptide sequences using their sequence logos (Fig. 5). Two important features of a multiple alignment that a sequence logo clearly illustrates are the "information content" at each alignment site and the "frequencies" of the nucleotides or amino acids visible there [60]. Thus, to understand the difference, the frequency of occurrence of amino acids at the cleavage site (positions 4 and 5) of our cleaved and un-cleaved octapeptide sequences were examined. In the case of cleaved octapeptide sequences, L is the most preferred amino acid at positions 4 and 5, while G and A are the least preferred at position 4 and T is the least preferred at position 5. In the case of un-cleaved octapeptide sequences, G and A are the most preferred at positions 4 and 5, respectively. In contrast, L is the least preferred at both positions 4 and 5. L was also the least preferred at the 2nd, 3rd, 6th, 7th, and 8th positions of the un-cleaved octapeptide sequences, considering the amino acid residues in the neighboring positions to positions 4 and 5. Similarly, in addition to A being the most preferred at position 5 of the un-cleaved octapeptide sequences, it was also the most preferred residue at positions 2, 3, 6, and 7. A pattern is clearly visible, with the most preferred amino acid residues in the cleaved octapeptide sequences being the least preferred in the un-cleaved ones. This is in consensus with the findings that L4 and L5 are among the top features selected by the GBC to make prediction on whether or not an octapeptide sequence is cleaved by the HIV-1 protease.

Summarily, it is worthy to note that our approach in this study is significantly different compared to previously reported studies covering similar niche. First, our method is quite exhaustive, instead of using various subsets of the benchmark datasets as training and validation sets [6, 16, 18–24], we concatenated the four subsets into one and thereafter performed various data wrangling processes involving the removal of duplicate and low variance entries, and dimensionality reduction. Then we went further to construct 178D descriptors consisting of amino acid binary profiles, standard physicochemical properties, and bond compositions. We applied a 3-way data split procedure on the dataset to generate the training set (70%)—for training the models and also for the 10-fold CV; validation set (10%)—for the models’ hyper-parameter tuning; and testing set (20%)—used as an external dataset for further evaluation of the models’ performance. Finally, we accounted for the imbalanced in our dataset by applying “stratified” 10-fold CV utilizing performance metrics such as *B. Acc.*, *AUC*, and *F-score* among others for our models’ evaluation and comparison. This way we avoided the complications of randomly distributing the positive and negative examples in our dataset into each fold, which may not be uniform.

## Conclusions

We have shown in this study that a combination of sequence information, including amino acid binary profiles, bond composition, and physicochemical properties can help identify HIV-1 protease cleavage sites and also throw light into the understanding of substrate specificity. Considering the comparable performances of the LR, MLPC, GBC, LDA, and NBC to that of the LSVM, we recommend the use of these models for the prediction of HIV-1 protease cleavage site. In the future, we shall make effort to develop the web-server and stand-alone software implementing the logistic regression and a hybrid of the constructed octapeptide descriptors discussed in this study as input variables for the prediction of HIV-1 protease cleavage site. Logistic regression was chosen because, unlike LSVM and other algorithms, it yields actual probabilities of the predicted class and is easy to update the model to take in fresh data.

## Supplementary Information

**Additional file 1: Fig. S1.** Confusion matrix of our models using the testing set.

**Additional file 2.** 178D octapeptide sequence descriptors calculated from Pfeature software.

## Data Availability

The methodology was implemented in Python programming language using Google Colaboratory/Jupyter-Notebook and the source code can be made available upon request from the corresponding author. Publicly available datasets were analyzed in this study. This data can be found here: https://archive.ics.uci.edu/ml/datasets/HIV-1+protease+cleavage. All other data generated during this investigation are included in this article.

## Abbreviations

HIV: Human immunodeficiency virus
AIDs: Acquired immunodeficiency syndrome
AABP: Amino acid binary profiles
MSA: Multiple sequence alignment
DTs: Decision trees
ANN: Artificial neural network
LSVM: Linear support vector machine
LDA: Linear discriminant analysis
GBC: Gradient boosting classifier
KNN: K-nearest neighbor
NBC: Naive Bayes classifier
DTC: Decision tree classifier
PC: Perceptron classifier
MLPC: Multi-layer perceptron classifier
LR: Logistic regression
TP: True positives
TN: True negatives
FP: False positives
FN: False negatives
TPR: True positive rate
TNR: False negative rate
ROC: Receiver operator characteristic curve
AUC: Area under the ROC curve
*FS*: F-score
*Sen.*: Sensitivity
*Spec.*: Specificity
*B. Acc.*: Balanced accuracy
*JI*: Jaccard-index
