# Supplementary figures and images for: Prediction of HIV-1 protease cleavage site from octapeptide sequence information using selected classifiers and hybrid descriptors
Source: BMC Bioinformatics. 2022 Nov 8;23:466. doi: 10.1186/s12859-022-05017-x (PMC9641908; doi:10.1186/s12859-022-05017-x)

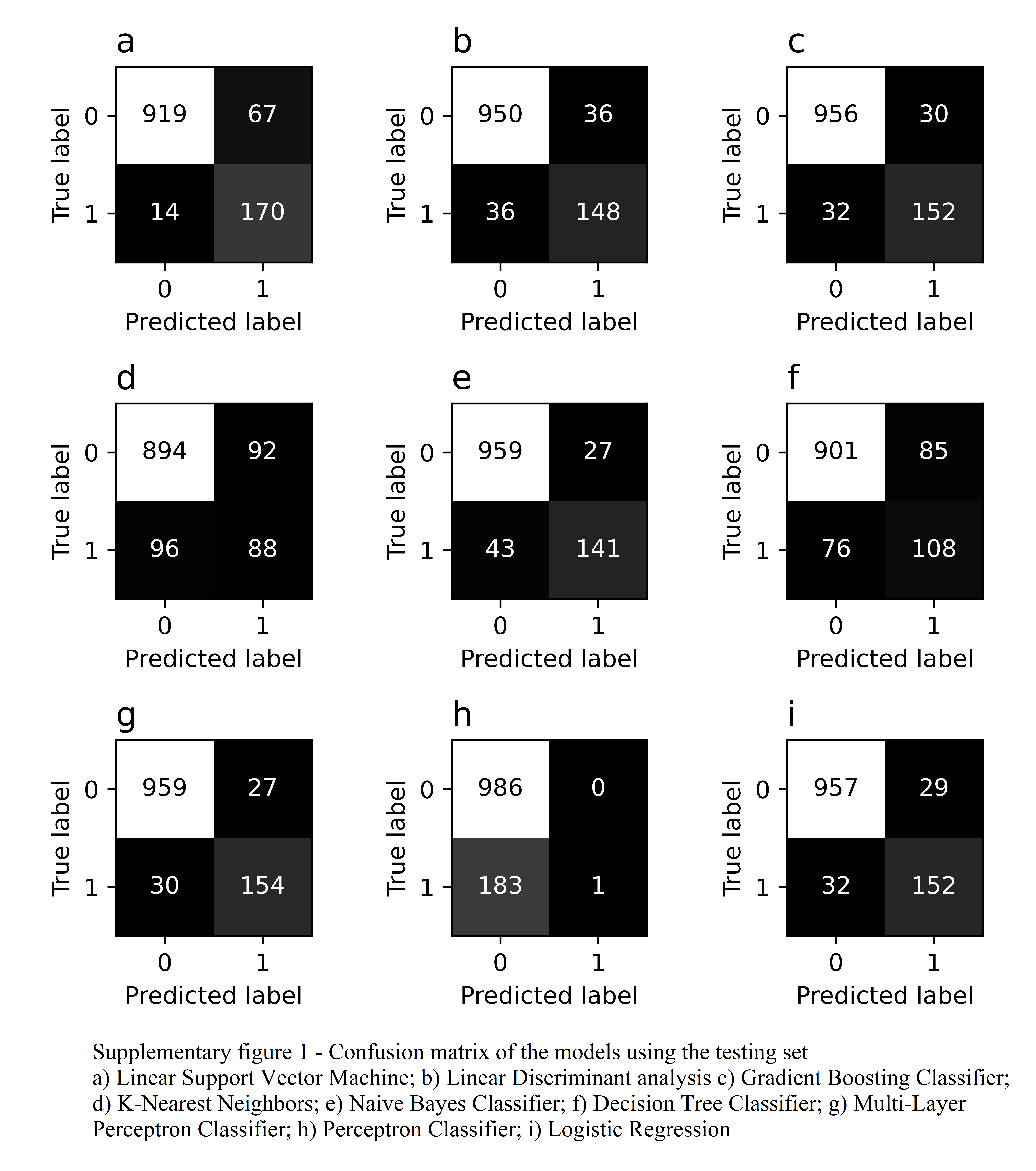

Supplement: Supplementary file 1 — Additional file 1: Fig. S1. Confusion matrix of our models using the testing set. [file 12859_2022_5017_MOESM1_ESM.png]
